# Does surgical or medical management of extrahepatic portosystemic shunts in dogs carry a better prognosis for the resolution and reduction of neurological dysfunction?

**DOI:** 10.18849/ve.v7i1.360

**Published:** 2022-02-09

**Authors:** Julia Smachlo+, Wanda Gordon-Evans+

**Keywords:** CANINE, CONGENITAL EXTRAHEPATIC PORTOSYSTEMIC SHUNT, MEDICAL MANAGEMENT, NEUROLOGICAL DYSFUNCTION, SURGICAL ATTENUATION

## Abstract

PICO question

In dogs with congenital extrahepatic portosystemic shunts that are treated with surgical attenuation what is the persistency, frequency, severity and outcome of neurological signs when compared to dogs that are treated medically?

Clinical bottom line

Category of research question

Prognosis

The number and type of study designs reviewed

Ten papers were critically reviewed

Strength of evidence

Weak

Outcomes reported

For short-term success, owners reported an overall decrease in neurological signs associated with neurological dysfunction and an increase in quality of life after the initiation of either medical management or surgical management. Surgical management has a weak association with higher mortality or severe neurological signs in the immediate postoperative period

Conclusion

It is challenging to make a direct comparison between medical and surgical management but, overall, both strategies seemed to decrease neurological signs in the short term. There was a lack of evidence and available data about the efficacy of each for long-term control of neurological signs

## How to apply this evidence in practice

The application of evidence into practice should take into account multiple factors, not limited to: individual clinical expertise, patient’s circumstances and owners’ values, country, location or clinic where you work, the individual case in front of you, the availability of therapies and resources.

Knowledge Summaries are a resource to help reinforce or inform decision making. They do not override the responsibility or judgement of the practitioner to do what is best for the animal in their care.

## Clinical scenario

Preanaesthetic bloodwork was consistent with a portosystemic shunt in a 6 month old male Yorkshire Terrier puppy. Subsequent bile acid stimulation tests were also consistent with a shunt. The owner would like to know what the prognosis and outcome of neurological dysfunction is assuming her dog has an extrahepatic portosystemic shunt (EHPSS) before she commits to referral for surgical attenuation compared to managing her dog medically without surgical intervention.

## The evidence

All of the studies analysed were retrospective studies, which have a higher level of bias than prospective studies. Furthermore, four of the retrospective studies were descriptive case series studies, which also have low levels of evidence. The remaining six studies were retrospective cohort studies, which have better evidence than descriptive studies, but overall still have low levels of evidence. Additionally, the majority of the studies did not evaluate the persistence, frequency and severity of neurological dysfunction following either surgery or medical management as their primary problem or did not make distinctions between intrahepatic and extrahepatic shunts, which made drawing conclusions from the data difficult. There was also very limited evidence on the efficacy of solely medical management for extrahepatic portosystemic shunts in dogs. Studies that directly compared the impact of surgical and medical management on portosystemic shunts did not differentiate between intrahepatic and extrahepatic shunts so they could not be made relevant to the question and were excluded.

## Summary of the evidence

**Favier et al. (2020):** 

Population:	Dogs with a single congenital portosystemic shunt (CPSS) confirmed by CT and / or ultrasound who did not have surgical intervention and received non-surgical treatment that were referred to Utrecht University, the Netherlands, between September 2003 and February 2015.
Sample size:	78 dogs.
Intervention details:	Case records were reviewed to identify dogs that received medical management for a single CPSS. 48 of these dogs had an extrahepatic CPSS and 29 had an intrahepatic CPSS. In one dog, CPSS was diagnosed, but the location of the shunt could not be established. Records for 65 dogs who were medically managed were divided in two treatment groups. One group (n = 27) was managed with restricted protein diet and lactulose for one month or until death. The other group (n = 38) was medically managed with only a restricted protein diet for 1 month or until death.
Study design:	Single centre retrospective cohort study.
Outcome studied:	Quality of life as assessed by the owner. Occurrence and frequency of clinical signs as assessed by the owner. Survival time. Presumed cause of death.
Main Findings (relevant to PICO question)	Estimated median survival time for dogs with an extrahepatic portosystemic shunts (EHPSS) was 41.5 months (95% CI 35.2–47.8). 20 EHPSS dog owners responded to the follow-up questionnaire (17 intrahepatic portosystemic shunt dog owners responded). 11 of these dogs received the adjusted diet and nine received the adjusted diet with lactulose. Of the 37 questionnaire responses, the CPSS scores were significantly lower (p = 0.01) with medical management (median 18) than before treatment (median 35). Of the 37 questionnaire responses, quality of life scores significantly increased (p<0.0001) with medical management (median 4) than before treatment (median 2).
Limitations:	Retrospective study is at risk for more bias than a prospective study. Subjective assessment of quality of life based on reporting. Reliance on owner recollection and follow-up, which increase the risk of recall bias and there was loss of follow-up with only 37/56 (66%) of the owners responding to the follow-up questionnaire. Owners elected euthanasia if quality of life declined, which could alter the accuracy of the estimated median survival time.

**Fryer et al. (2011):** 

Population:	Dogs with a single extrahepatic portosystemic shunt (EHPSS) confirmed by abdominal ultrasounds, nuclear scintigraphy, or clinically relevant bile acids or ammonia concentration, and underwent surgery application of an ameroid constrictor at Texas A&M University’s Veterinary Medical Teaching Hospital, Texas, USA, between January 2003 and November 2010.
Sample size:	126 dogs.
Intervention details:	Electronic medical records were searched to find eligible dogs for the study. Surgical management of the EHPSS with an ameroid constrictor. Dogs were divided into two groups, 42 dogs who were treated with levetiracetam (LEV) at a median of 60 mg/kg/day at least 1 day before EHPSS ligation, and 84 dogs that had no anticonvulsant drugs before EHPSS ligation.
Study design:	Single centre retrospective cohort study.
Outcome studied:	Proportion of dogs with neurological dysfunction. Blood ammonia concentrations. Postoperative complications.
Main Findings (relevant to PICO question)	4/84 dogs (5%) in the group that did not receive LEV had postoperative seizures. Risk of seizures was significantly (p<0.0002) <1 for the dogs that received LEV with Bayesian statistics. 4/126 dogs (3%) postoperatively had generalised motor seizures and did not survive to discharge.
Limitations:	Retrospective study is at risk for more bias than a prospective study. No follow-up past the hospital discharge with the dogs to assess additional postoperative neurological dysfunction.

**Harvey & Erb (1998):** 

Population:	Dogs with a congenital extrahepatic portosystemic shunt (CEPSS) that presented to the Veterinary Medical Teaching Hospital of the College of Veterinary Medicine at Cornell University, USA, between 1985 and 1996.
Sample size:	56 dogs.
Intervention details:	Medical records were reviewed to identify dogs with complete records that underwent surgery for a CEPSS. Surgical management of the CEPSS with complete or partial ligation.
Study design:	Single centre retrospective case series.
Outcome studied:	Preoperative signs of hepatic encephalopathy including: transient blindness, circling, seizures, head pressing, and disorientation. Ability to completely ligate the single shunting vessel in non-encephalopathic dogs compared to encephalopathic dogs. Follow-up of neurological signs in non-encephalopathic dogs.
Main Findings (relevant to PICO question)	12/56 dogs (21%) displayed no preoperative signs of encephalopathy and 44/56 dogs (79%) displayed preoperative signs of encephalopathy. 1–10 year follow-up period of 8/12 non-encephalopathic dogs reported the development of no neurological signs as reported by the owners.
Limitations:	Retrospective study is at risk for more bias than a prospective study. Case series is at risk for more bias and relies heavily upon accurate medical records and clear decision-making processes noted in the record. There was no follow-up for the 44 dogs that had preoperative signs of encephalopathy. Loss of data (4/12 dogs) in the follow-up period with the non-encephalopathic dogs. The follow-up period for the non-encephalopathic dogs was vague and did not indicate how many dogs made it to the 10–year follow-up, or if most of the owners had only provided short-term (1 year) follow-up data. Poor data provided about the long-term prognosis. The owners did not report the presumptive cause of death in their dogs. Reliance on owners to report neurological signs for the follow-up period increases risk of error because owners might miss subtle neurological signs.

**Hunt & Hughes (1999):** 

Population:	49 dogs that underwent surgical attenuation using silk suture at the Veterinary Cardiovascular Unit, Department of Veterinary Clinical Sciences, The University of Sydney, Australia, between 1989 and 1997.
Sample size:	49 dogs.
Intervention details:	Medical records were reviewed to identify dogs that underwent surgical attenuation using silk suture for an extrahepatic portosystemic shunt. Surgical management of the congenital extrahepatic portosystemic shunt with silk suture attenuation and complete or partial ligation.
Study design:	Single centre retrospective case series.
Outcome studied:	Clinical signs and problems developed during the perioperative (first 24 hours postoperatively), early postoperative (24 hours postoperatively – skin suture removal) and long-term periods. Clinical efficacy of the primary surgeon (38 cases) compared to one of six other surgeons. Degree of shunt attenuation. Portal pressure post-ligation. Biochemical lab data obtained postoperative (2 weeks to 6 years).
Main Findings (relevant to PICO question)	48/49 dogs survived to skin suture removal. 1/49 (2%) mortality rate. Five dogs experienced neurological abnormalities (seizures or ataxia) suspected to be a manifestation of post-ligation seizure syndrome 9/49 dogs (18%) displayed neurological signs in the early postoperative period, seven (14%) of those were considered to be related to the surgical attenuation, and two were due to hypoglycaemia. Six were ataxic, two had generalised motor seizures, one was disoriented and one had both ataxia and muscle tremors. Three dogs that developed ataxia (without hypoglycaemia or hyperammonemia) within 1 week of surgery resolved without medical treatment. Two dogs with ataxia were hypoglycaemic and ataxia resolved with glucose administration. One of the dogs with seizures in the early postoperative period was refractory to phenobarbital and died within 24 hours. One dog developed ataxia, muscle tremors, and portal hypertension leading to shunt ligature removal. 9/45 dogs (20%) had recurrence of hepatic encephalopathy within 18 months postoperatively. One was due to development of acquired shunts. In the others, patency of the original shunting vessel was suspected.
Limitations:	Reliance on owners to report neurological signs for the follow-up period increases risk of error because owners might miss subtle neurological signs. Retrospective study is at risk for more bias than a prospective study. Case series is at risk for more bias and relies heavily upon accurate medical records and clear decision-making processes noted in the record. Difficult to differentiate in the perioperative period whether the majority of the neurological signs in the dogs were attributed to post-ligation neurological dysfunction or had contributing components of hypoglycaemia and/or hyperammonemia. The follow-up with owners was relatively short-term to assess continued neurological dysfunction.

**Mullins et al. (2019):** 

Population:	Dogs that underwent surgical attenuation of a single congenital extrahepatic portosystemic shunt (EHPSS) from January 2005 to July 2017 at 10 veterinary institutions and developed post-attenuation seizures (PAS) within 7 days postoperatively.
Sample size:	940 dogs.
Intervention details:	Medical records were reviewed to identify dogs that underwent surgical attenuation of a single congenital EHPSS. Surgical management of congenital EHPSS with attenuation. Dogs were divided into three groups. One group of 523 dogs did not receive levetiracetam (LEV). The second group of 188 dogs received LEV at a dose greater than 15 mg/kg every 8 hours for more than 24 hours preoperatively or a 60 mg/kg IV loading dose perioperatively with continuation of LEV postoperatively at greater than 15 mg/kg every 8 hours. The third group of 229 dogs received less than 15 mg/kg every 8 hours for less than 24 hours preoperatively or LEV postoperatively at less than 15 mg/kg every 8 hours.
Study design:	Multi-centre retrospective cohort study.
Outcome studied:	Development of PAS within 7 days postoperatively between the three groups. Short term survival (30 days postoperatively) for the dogs that developed PAS.
Main Findings (relevant to PICO question)	75/940 dogs (7%) developed PAS within 7 days postoperatively. 62 had generalised PAS and 13 had focal PAS. Seizures onset at a median of 48 hours (range 8–128 hours) postoperatively. 61/75 dogs (81%) that developed PAS had preoperative neurological signs including: lethargy, pacing/compulsive walking, dullness, head pressing, ataxia, abnormal change in behavior, hypersalivation/drooling, circling, etc. 11/75 dogs (15%) that developed PAS had preoperative seizures. 35/75 PAS dogs (6 were in the first group (6.7%), 21 of the dogs were in the second group (11.2%), and 19 dogs were in the third group (8.3%). The difference between groups was not significant (p = 0.14). 74/75 PAS dogs received preoperative medical management including a restricted protein diet, lactulose and antimicrobial therapy. Ammonia and glucose concentrations were normal for the30 and 36 PAS dogs, respectively. 23 of the 75 PAS dogs survived to 30 days postoperatively.
Limitations:	Retrospective study is at risk for more bias than a prospective study. The 75 PAS dogs were reported to have received preoperative medical management and it is unclear if this was continued after surgery. Difficult to discern the impact of strictly surgical intervention. Only had access to 30 and 36 of the 75 PAS dogs’ serum ammonia and glucose concentrations respectively, so neurological dysfunction caused by PAS and hepatic encephalopathy cannot be differentiated. There was no follow-up of the dogs that did not display signs of PAS within 7 days postoperatively. There was no long-term follow-up of the PAS dogs.

**Strickland et al. (2018):** 

Population:	Dogs that underwent partial or complete surgical attenuation of a single congenital portosystemic shunt (CPSS) confirmed by a mesenteric portovenogram at one institution between February 2000 and July 2015.
Sample size:	253 dogs.
Intervention details:	Medical records were reviewed to identify dogs that underwent surgical attenuation of a single CPSS. 196 dogs had an extrahepatic shunt and 57 had an intrahepatic shunt. Surgical management of CPSS. 238/253 dogs (94%) received medical management prior to surgery. Dogs with CPSS often display signs of hepatic encephalopathy (HE), which may be linked to the development of post-attenuation neurological signs (PANS), a complication observed with surgical management of CPSS.
Study design:	Single centre retrospective cohort study.
Outcome studied:	Proportion of dogs that developed PANS. Proportion of dogs that had postoperative seizures. Proportion of dogs that had preoperative and postoperative HE. Duration of clinical signs preoperative. Duration and response to medical management preoperative. Proportion of dogs with preoperative HE that developed PANS.
Main Findings (relevant to PICO question)	57 days was the median duration of clinical signs of HE prior to surgery. 176/238 dogs (74%) with HE preoperatively responded well to medical management, 49/238 (21%) responded moderately, and 13/238 (5%) responded poorly. 23/196 (12%) dogs with an extrahepatic shunt developed PANS. 10 dogs with an extrahepatic shunt had postoperative seizures. 22/28 dogs (79%) that developed PANS had preoperative signs of HE before surgery. 9/28 dogs (32%) had signs of HE immediately before surgery. 23/28 dogs had an extrahepatic shunt and 5/28 dogs had an intrahepatic shunt. Presence of HE preoperatively increased the odds of PANS 2.704-fold. There was no association between shunt location and incidence of PANS or seizures.
Limitations:	Retrospective study is at risk for more bias than a prospective study. There was no long-term follow-up. The study did not separate intrahepatic and extrahepatic preoperative HE and severity of PANS and seizures.

**Tisdall et al. (2000):** 

Population:	Dogs with a congenital portosystemic shunt (CPSS) that presented to the University Veterinary Centre, Sydney, Australia, between August 1989 and February 1999.
Sample size:	121 dogs.
Intervention details:	Medical records were reviewed to identify dogs with a CPSS. 89 dogs had extrahepatic shunts and 32 had intrahepatic shunts. Surgical management of the extrahepatic shunts: 12 were complete attenuation and 77 were partial attenuation. 31 dogs who had surgery after June 1997 received prophylactic phenobarbital (5–10 mg/kg) and for 2 weeks (3–5 mg/kg q12) postoperatively.
Study design:	Single centre retrospective cohort study.
Outcome studied:	Neurological dysfunction of dogs with a congenital extrahepatic shunt within 7 days postoperatively. Serum phenobarbitone levels in dogs with a congenital extrahepatic shunt treated prophylactically with phenobarbitone.
Main Findings (relevant to PICO question)	70/89 dogs (79%) displayed signs of hepatic encephalopathy preoperatively. Seven of these dogs displayed neurological dysfunction postoperative, and one had seizures preoperative that did not resolve postoperative. 11/89 dogs (12%) displayed neurological dysfunction within 7 days postoperatively. Three of these dogs did not display neurological signs preoperatively. Nine of the dogs did not receive phenobarbitone preoperatively. Three dogs developed seizures that lead to status epilepticus and were initially refractory to therapy. One succumbed to cardiac arrest. The other two responded to thiopentone or pentobarbitone and were maintained on phenobarbitone. Three dogs had nonprogressive ataxia which resolved without treatment. Three dogs had partial seizures with the reduction of postoperative phenobarbitone doses. Four dogs had severe neurological dysfunction, but no seizures. Two were receiving phenobarbitone and responded to additional phenobarbitone. The other two, which had an abnormal anaesthetic recovery, responded to phenobarbitone and acepromazine. One resolved within 72 hours. One was blind and ataxic at discharge 15 days postoperatively and had a cellophane band placed 8 weeks later which resolved the neurological signs but not the blindness. One had residual hypermetria at discharge 14 days postoperatively, which resolved in 1 week. One had residual ataxia and central blindness 14 days postoperatively, with the ataxia resolving 4 weeks postoperatively and the blindness improving over 2 months. None of the 31 dogs who received phenobarbitone prophylactic had seizures, but two were ataxic. Serum phenobarbitone levels were in therapeutic ranges for the seven dogs assessed 3 days postoperatively.
Limitations:	This was a retrospective case series, which has a low level of evidence and greater risk of bias than a prospective study. Short follow-up period at seven days postoperatively. This might not be reflective of the full risk and cases/signs that developed or severely persisted past 7 days. Small sample size.

**Wallace et al. (2018):** 

Population:	Pugs undergoing surgical attenuation for a single congenital extrahepatic portosystemic shunt (CEPSS) and had at least 1 month of postoperative follow-up at Colorado State University Veterinary Teaching Hospital, Colorado, USA, between 1998 and 2010. Various breed dogs (not Pugs) undergoing surgical attenuation for a single CEPSS and had at least 1 month of postoperative follow-up at Colorado State University Veterinary Teaching Hospital between 1997 and 2005.
Sample size:	44 dogs.
Intervention details:	Reviewed medical records to identify Pugs and control dogs with a single CEPSS in the study. Surgical management of the single CEPSS. 14 Pugs were the study group and 30 other breeds of dogs were in the control group.
Study design:	Single centre retrospective cohort study.
Outcome studied:	Preoperative information including the signalment and clinicopathologic data. Postoperative neurological signs, specifically seizures. Postoperative mortality and complications. Bile acids measurement. Date and cause of death.
Main Findings (relevant to PICO question)	4/14 Pugs (29%) were euthanised within 1 month of surgery from continued and/or worsening neurological signs. Two were euthanised due to intractable seizures and two were euthanised because of worsening and severe neurological signs between 2 and 29 days postoperatively. There was no evidence of necrotising meningoencephalitis. One of the control group dogs died from intractable seizures within 1 month postoperatively, which was significantly lower mortality than the Pug group postoperatively. 6–72 month follow-up of 9/10 Pugs that survived. The owners found no neurological signs or need for medical management. Bile acids were decreased postoperatively.
Limitations:	The study is a retrospective study, which has more bias than a prospective study and relies on very accurate medical records and follow-up with owners. There was a loss of follow-up data with some of the Pugs postoperatively. Small sample size. All the dogs in the study did not undergo the same surgical procedure, most underwent cellophane banding, and one underwent ameroid constrictor placement, which introduces bias because one surgical methodology may be more effective than the other. Differences between the dates of study for the Pugs and control group, as well as the significantly smaller average dog in the control group compared to the Pugs.

**Watson & Herrtage (1998):** 

Population:	Dogs with a congenital portosystemic shunt (CPSS; confirmed by ultrasound, contrast radiography, surgery or postmortem) and were only managed medically that presented to the Department of Clinical Veterinary Medicine (DCVM), University of Cambridge, UK, between January 1987 and December 1993.
Sample size:	27 dogs.
Intervention details:	Case records were reviewed to identify eligible dogs for inclusion in the study. Nine dogs had extrahepatic shunts, 17 had intrahepatic shunts and one dog had a complex partially intrahepatic and partially extrahepatic shunt. Dogs were managed medically. 23 dogs were fed a reduced protein diet (Canine u/d or kd/; Hill’s Pet Products; homemade or a combination). Two dogs were fed an unknown brand of proprietary dog food with normal protein levels. Two dogs had no diet recorded. 24 dogs received lactulose, consistently for 18/24 dogs (75%) and at the start of treatment for 6/24 dogs (25%). 2/27 dogs (7%) did not receive lactulose and 1/27 dogs (4%) had no record. Antibiotics were administered to 24/27 dogs (89%). 2/27 dogs (7%) did not receive antibiotics and 1/27 dog (4%) had no record.
Study design:	Single centre retrospective case series.
Outcome studied:	Clinical signs, signalment, and blood work at initial presentation. Change in clinical signs reported by owner after initiation of medical management. Follow-up with owners 3 years after end of study for mortality assessment.
Main Findings (relevant to PICO question)	7/9 dogs (78%) with EHPSS had owner-reported neurological signs (hyperactivity, depression head-pressing, circling, excessive salivation, confusion, coma) prior to presentation. Two dogs with EHPSS were lost to follow-up. 1/7 dogs (14%) were reported by the owner to have neurological signs more often after the start of medical management. 3/7 dogs (43%) were reported by the owners to have neurological signs the same after the start of medical management. 3/7 dogs (43%) were reported by the owners to have neurological signs less frequently after the start of medical management. 3 years after study (October 1996), three dogs with extrahepatic shunts had been euthanised due to uncontrollable neurological signs or ascites. 3/9 dogs (33%) with extrahepatic shunts were still alive 3 years after the end of the study.
Limitations:	Small sample size overall, but particularly for dogs with an extrahepatic CPSS. Loss of information with owner follow-up, only able to connect with 14 cases. Unclear how long initial follow-up was after initiating medical management. Unclear which dogs received what components of the medical management, if some received all three and if some dogs received none of the three. Subjective assessment in owner follow-up could lead to increased risk of bias because not all owners interpret their dog’s change in clinical signs the same. The subjective assessment also did not include severity of the clinical signs noted.

**Worley & Holt (2008):** 

Population:	Dogs over five years old with a single congenital extrahepatic portosystemic shunt (EHPSS) that underwent surgical attenuation presenting at the University of Pennsylvania, USA, between 1992 and 2005.
Sample size:	17 dogs.
Intervention details:	Medical records were reviewed to identify dogs eligible for inclusion in the study. Surgical management of EHPSS: six dogs underwent complete shunt ligation and 11 underwent partial shunt attenuation.
Study design:	Single centre retrospective case series.
Outcome studied:	Preoperative data on the dogs were collected including signalment, clinical signs, and clinicopathologic findings. Postoperative mortality rate and cause of death. Postoperative complications and clinical signs from reevaluations and follow-up with owners and referring veterinarians. Postoperative liver-function tests.
Main Findings (relevant to PICO question)	12/17 dogs (71%) preoperatively displayed neurological signs which could include abnormal mentation, ataxia, and seizures. Immediate postoperative mortality rate was 2/17 dogs (12%). One dog with preoperative neurological signs developed intractable seizures that were unresponsive to multimodal drug therapy and was euthanised 1 week after surgery. One dog went into cardiac arrest shortly after surgery. Three dogs with preoperative neurological signs developed minor neurological signs post-surgery and two required short-term therapy. 4/17 dogs (24%) displayed neurological signs postoperatively.
Limitations:	The study had a small sample size. This was a retrospective case series, which has a low level of evidence and greater risk of bias than a prospective study. The study assumes that the record keeping was accurate. Postoperative data were collected through reevaluations and telephone follow-up with owners and referring veterinarians, which are subject to increased bias with two of the 15 discharged dogs data lost to follow-up.

## Appraisal, application and reflection

One of the most common clinical signs for a dog at presentation with an extrahepatic portosystemic shunt (EHPSS) is neurological signs, which can range in severity from mild head pressing and ataxia to seizures. The majority of EHPSSare managed through surgical attenuation, but that is not always an option for owners or achievable due to the anatomy of the shunt. Preoperative neurological signs of hepatic encephalopathy secondary to congenital extrahepatic portosystemic shunt (CEPSS) is caused predominantly by hyperammonemia, which decreases following successful shunt attenuation. The central nervous system may experience chronic astrocyte changes from a CEPSS, which causes an abnormal metabolic environment preoperatively and the sudden change of surgical attenuation favours an excitatory state. Post-attenuation neurological signs (PANS) is suspected to develop post attenuation when a metabolic event occurs in conjunction with the chronic preoperative central nervous system changes. Other contributing factors for dogs developing PANS is unknown, with conflicting evidence about the association of increasing age and shunt attenuation method. Unlike with hepatic encephalopathy, dogs that experience PANS have normal ammonia levels. There were no studies that had a direct comparison between the effectiveness of surgical or medical management of EHPSS in decreasing neurological signs. Overall, Strickland et al. (2018) found the lowest incidence of short-term postoperative neurological signs at 4/126 dogs (3%), while Wallace et al. (2018) found the highest incidence of short-term postoperative neurological signs at 4/14 dogs (29%) and Watson & Herrtage (1998) found an incidence of owner-reported decrease in neurological signs after the initiation of medical management of 3/7 dogs (43%), as well as a reported increase in quality of life.

Favier et al. (2020), Watson & Herrtage (1998), and Strickland et al. (2018) found that the owners reported a decrease in neurological signs and an increase in quality of life after the initiation of medical management. However, these studies lost several dogs in the follow-up period and had relatively small sample sizes. Watson & Herrtage (1998) also evaluated the long-term efficacy of medical management and at 3 years found that only 3/9 dogs (33%) of the dogs included in the follow-up were still alive.

All the studies that evaluated for surgical management found that there was an overall decrease in neurological signs from preoperatively to postoperatively. Worley & Holt (2008) found that 12/17 dogs (71%) in their study displayed preoperative neurological signs, which decreased to 4/17 dogs (24%) after surgical attenuation. However, there was a risk of perioperative mortality or dogs succumbing to refractory seizures with 48 hours postoperatively. For instance, Tisdall et al. (2000) found that with surgical attenuation, the overall proportion of dogs with neurological signs decreased from 70/89 (79%) preoperatively to 11/89 (12%) postoperatively. The majority of the dogs with signs postoperatively had also displayed neurological signs prior to surgery. The study also found that there is a very weak association with the use of phenobarbital to decrease the incidence of postoperative seizures. Only two of the studies evaluated the longer-term efficacy of surgical attenuation in decreasing neurological signs associated with EHPSS. Wallace et al. (2018) had a follow-up ranging from 6 to 72 months postoperatively in nine Pugs with owners reporting no neurological signs or need for medical management. Harvey & Erb (1998) had a 1–10 year follow-up period of eight of the 12 non-encephalopathic dogs, and owners reported the development of no neurological signs. However, both of the longer-term evaluations were based on owner reports, they were small sample sizes, and it is unclear how many dogs actually made it very far into the range of the follow-up period.

The studies suggest that there may be a weak association with higher mortality and/or increased severity of neurological signs that results in euthanasia in the immediate perioperative period for surgical management than after the initiation of medical management. For example, Fryer et al. (2011) had four dogs in the immediate postoperative period that had generalised motor seizures and did not survive to hospital discharge. Similarly, Wallace et al. (2018) found that four of the 14 Pugs were euthanised within 1 month of surgery from continued and/or worsening neurological signs and intractable seizures, and one of the control group dogs died from intractable seizures within 1 month postoperatively. However, although no immediate deaths were reported in either of the studies that addressed medical management, it cannot be ruled out because there is no clearly defined timeline for the dogs’ deaths.

Two of the biggest limitations with these retrospective studies is that many only had very short-term follow-up periods and the data of several dogs were lost in the follow-up period. It was difficult to discern how a dog’s quality of life might be impacted 5 years down the road after the initiation of medical management or postoperatively. Additionally, the owners were predominantly relied upon for accurate observation and reporting of neurological signs in their dogs. However, some neurological signs can be very subtle and might have been missed by the owner.

Further research should include prospective studies that have a clearly defined definition and inclusion criteria for post-attenuation neurological syndrome including blood ammonia and glucose levels postoperatively.

## Methodology Section

**Search Strategy:** 

Databases searched and dates covered:	CAB Abstracts on OVID Platform; January 1973–May 2020 PubMed on NCBI Platform; January 1987–May 2020
Search strategy:	CAB Abstracts: (extrahepatic portosystemic shunt OR EHPSS OR CEPSS) AND (dog OR canine) AND (neurologic OR neurological OR seizure OR hepatic encephalopathy) PubMed: (congenital extrahepatic portosystemic shunt OR EHPSS OR CEPSS) AND (dog OR canine) AND (neurologic OR neurological OR seizure OR hepatic encephalopathy)
Dates searches performed:	28 Feb 2021

**Exclusion / Inclusion Criteria:** 

Exclusion:	Book chapters, articles not available in English, clinical review articles, proceedings.
Inclusion:	Articles available in English that were relevant to the PICO, articles had to involve more than five dogs.

**Search Outcome:** 

Database	Number of results	Excluded – Clinical review article	Excluded – Not relevant to PICO	Excluded – Case reports with less than five dogs	Excluded – Full article not available	Total relevant papers
CAB Abstracts	8	0	5	1	0	2
PubMed	26	2	11	3	1	9
Total relevant papers when duplicates removed						10

## Conflict of Interest

The authors declare no conflicts of interest.

## Disclaimer

Knowledge Summaries are a peer-reviewed article type which aims to answer a clinical question based on the best available current evidence. It does not override the responsibility of the practitioner. Informed decisions should be made by considering such factors as individual clinical expertise and judgement along with patient’s circumstances and owners’ values. Knowledge Summaries are a resource to help inform and any opinions expressed within the Knowledge Summaries are the author’s own and do not necessarily reflect the view of the RCVS Knowledge. Authors are responsible for the accuracy of the content. While the Editor and Publisher believe that all content herein are in accord with current recommendations and practice at the time of publication, they accept no legal responsibility for any errors or omissions, and make no warranty, express or implied, with respect to material contained within. For further information please refer to our Terms of Use.
